# Synthesis, Structure, Crystallization and Mechanical Properties of Isodimorphic PBS-*ran*-PCL Copolyesters

**DOI:** 10.3390/polym13142263

**Published:** 2021-07-09

**Authors:** Maryam Safari, Itziar Otaegi, Nora Aramburu, Gonzalo Guerrica-Echevarria, Antxon Martínez de Ilarduya, Haritz Sardon, Alejandro J. Müller

**Affiliations:** 1 POLYMAT and Department of Polymers and Advanced Materials: Physics, Chemistry and Technology, Faculty of Chemistry, University of the Basque Country UPV/EHU, Paseo Manuel de Lardizabal, 3, 20018 Donostia-San Sebastián, Spain; maryam.safari@polymat.eu (M.S.); itziar.otaegi@ehu.eus (I.O.); nora.aramburu@ehu.eus (N.A.); gonzalo.gerrika@ehu.eus (G.G.-E.); haritz.sardon@ehu.eus (H.S.); 2 Departament d’Enginyeria Química, L’Escola Tècnica Superior d’Enginyeria Industrial de Barcelona (ETSEIB), Universitat Politècnica de Catalunya, Diagonal 647, 08028 Barcelona, Spain; antxon.martinez.de.ilarduia@upc.edu; 3 IKERBASQUE, Basque Foundation for Science, Plaza Euskadi 5, 48009 Bilbao, Spain

**Keywords:** isodimorphism, PBS-*ran*-PCL, random copolymers, crystallization

## Abstract

Isodimorphic behavior is determined by partial inclusion of comonomer segments within the crystalline structure and arises from the comparatively similar repeating chain units of the parental homopolymers. Isodimorphic random copolymers are able to crystallize irrespective of their composition and exhibit a pseudo-eutectic behavior when their melting point values are plotted as a function of comonomer content. At the pseudo-eutectic point or region, two crystalline phases can coexist. On the right-hand and the left-hand side of the pseudo-eutectic point or region, only one single crystalline phase can form which is very similar to the crystalline structures of the parent homopolymers. This article aims to study the synthesis method, structure, crystallization behavior and mechanical properties of isodimorphic random PBS-*ran*-PCL copolyesters. Moreover, this study provides a comprehensive analysis of our main recent results on PBS-*ran*-PCL random copolyesters with three different molecular weights. The results show that the comonomer composition and crystallization conditions are the major factors responsible for the crystalline morphology, crystallization kinetics and mechanical performance of isodimorphic random copolyesters. Our studies demonstrate that in the pseudo-eutectic region, where both crystalline phases can coexist, the crystallization conditions determine the crystalline phase or phases of the copolymer. The relationships between the comonomer composition and mechanical properties are also addressed in this work.

## 1. Introduction

Polymers constitute basic structural materials that participate in vital life processes due to their applications and demands in diverse areas of science and industry. However, the concern about serious environmental crises caused by fossil sources has driven the research of biodegradable and biobased polymers [1,2,3,4,5]. Aliphatic polyesters are developing as an important branch of biodegradable and biocompatible polymers for uses in many diverse fields including coatings, drug delivery processes, packaging, tissue engineering and many more. Each of these applications seeks materials with specific chemical, physical, mechanical and degradation properties [6,7,8,9,10].

Unfortunately, these kinds of biopolymers do not usually meet all the mechanical requirements needed for specific applications and they are also characterized by a high degree of crystallinity which limits their biodegradation rate. This problem is often addressed by copolymerizing two different types of monomers. Copolymerization makes it possible to have a better control over the structures than by blending homopolymers. Moreover, the composition can be varied over a wide range, and molecular weight can be controlled to achieve the desired properties in the final product [11,12,13].

Among biodegradable polyesters, Poly (ε-caprolactone) (PCL) has been widely considered as a good candidate for biomedical and packaging uses. PCL is a soft biocompatible and biodegradable semi-crystalline polyester that has a low melting point (about 55–70 °C) and a low glass transition temperature (around −60 °C). However, the mechanical properties and biodegradability of the neat PCL do not meet some of the demands of biomedical fields such as bone tissue engineering [14,15]. Poly(butylene succinate) (PBS) has a higher melting point than PCL (∼120 °C), good flexibility, toughness, thermal and chemical resistance [16,17].

**Figure 1:**
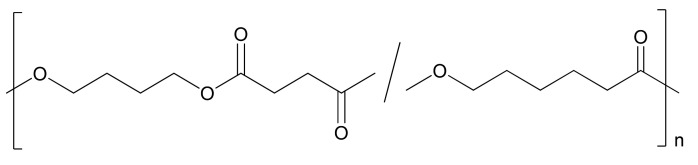
Chemical structure of PBS-*ran*-PCL copolyesters.

Random copolymerization is a common and beneficial method of combining the desired properties of two different homopolymers in a single copolymer in order to tailor their biodegradation rates by controlling the crystallinity degree. Moreover, copolymerization can restrict the degree of copolymer crystallization and enhance their physical properties to expand their applications and meet the biodegradable polymer demand. The magnitudes of properties such as crystallinity, flexibility, crystallization and melting temperatures and the glass transition temperature change, and even the directions of these changes can be selected depending on composition. In contrast with the blending of immiscible polymers, random copolymerization creates random covalent bonds between two parent comonomers, leading to full melt miscibility. Merging PCL properties such as softness, flexibility, biocompatibility with those of PBS, can result in a material that can be used in a wider range of applications. The chemical structures of PBS-*ran*-PCL random copolyesters are shown in Figure 1.

There are three main behaviors in which random copolymers can crystallize which strongly depend on the molecular weight, molar ratio and most importantly, the chemical structure of the repeating units of the copolymer.

(1) Total inclusion behavior: It happens when the two comonomeric units contain similar repeating units. The chains can co-crystallize regardless of the composition, the so-called isomorphic behavior. In this case, the two comonomers are miscible in the crystalline state, and thermal and structural properties generally show a linear dependence on composition [18].
(2) Inclusion-exclusion behavior: When the two homopolymers have similar repeating units, but they do not share any crystalline structure, an isodimorphic behavior in the random copolymers can result. This group of random copolymers shows crystallization over the whole composition range and a pseudo-eutectic behavior characterized by a dominant crystalline structure at each side of the pseudo-eutectic point or region. Moreover, the changes in the unit cell parameters of both crystal phases with composition evidence comonomer inclusion. Our group has recently studied isodimorphism in several random copolymer systems [19,20,21,22,23,24,25,26,27,28,29]. Isodimorphic crystallization has recently been found in different types of random copolymers such as poly (decamethylene succinate-*ran*-decamethylene fumarate), poly (butylene succinate-co-cis-butene succinate) and poly (hexamethylene carbonate)-co-poly (hexamethylene urethane) [30,31,32,33,34,35].
(3) Total exclusion behavior: When the repeating units of two homopolymers are dissimilar, the minor comonomer would be completely kept out from the crystalline structure of the main component. Therefore, the transition temperatures and enthalpies decrease significantly with increasing comonomer content and the copolymer remains completely amorphous over a large range of copolymer compositions [19].

In this paper, biodegradable poly(butylene succinate-*ran*-caprolactone) [PBS-*ran*-PCL] copolyesters with a higher molecular weight than those produced in our earlier works were synthesized successfully. This paper also reviews the main results of our recent research on the synthesis, crystalline structure, crystalline morphology and crystallization kinetics of PBS-*ran*-PCL. In order to study the effect of comonomer incorporation on the mechanical properties of isodimorphic random copolymers, tensile and DMTA tests were also performed.

## 2. Materials and Methods

### 2.1. Materials

1,4-butanediol (BD), ε-caprolactone (CL), dimethyl succinate (DMS) and titanium tetraisopropoxide (TTP)—as the catalyst—were purchased from Aldrich and were used as received.

### 2.2. Synthesis Routes of BS_x_CL_y_ Random Copolyesters

The first series of copolyesters were successfully synthesized through enzyme-catalyzed ring-opening polymerization (ROP) of butylene succinate cyclic oligomers and ε-caprolactone using lipase B from *Candida antarctica* (CALB) as a catalyst. Copolyesters obtained by these copolymerizations are named as LM_W_-BS_x_CL_y_. Weight average molecular weights of these copolyesters were between 4k and 14k g/mol and LM_W_ denote them as low molecular weight materials in comparison with other series prepared later. LM_W_-BS_x_CL_y_ were studied in detail in one of our previous works (see ref. [36] for more details).

As the molecular weight of copolyesters produced by enzymatic ROP is strongly affected by polymerization conditions, such as the presence of small traces of water molecules and low reaction temperatures, the second series of copolyesters were synthesized by a metal-catalyzed melt-polymerization in the presence of titanium tetraisopropoxide (TTP) as the catalyst in order to increase the molecular weight [37,38]. In summary, the transesterification-ROP reactions of ε-caprolactone (CL), 1,4-butanediol (BD) and dimethyl succinate (DMS) were carried out at 160 °C for 4 h, and then the polymerizations were completed by polycondensation at reduced pressure and 190 °C for 3 h. We named this series of copolyesters MM_W_-BS_x_CL_y_ (i.e., medium molecular weights series). These copolyesters were studied in detail in our previous research works (see refs. [37,38]). The weight-average molecular weights of MMw-BS_x_CL_y_ copolyesters were between 17k−30k g/mol. The M_W_ of MM_W_-BS_x_CL_y_ copolyesters were close to 4 times higher than those of the first series of copolyesters synthesized by enzymatic ROP copolymerization (named LM_W_-BS_x_CL_y_).

In this work, the third series of PBS-*ran*-PCL copolymers were synthesized in order to obtain very high molecular weight copolyesters. Hence, by increasing the polycondensation time (to 6 h) and the temperature (to 220 °C), we obtained high molecular weight copolyesters (named HMw-BS_x_CL_y_) (see Table S1). The weight average molecular weights of the HM_W_-BS_x_CL_y_ copolyesters are in the range of 40k–90k g/mol and are around 10 times higher than those of the first series of copolyesters synthesized by enzymatic ROP copolymerization (LM_W_-BS_x_CL_y_).

As a result, three series of PBS-*ran*-PCL random copolymers were synthesized successfully over a wide range of compositions by changing the polycondensation conditions so as to obtain different molecular weights. The copolyesters synthesized for this research work were abbreviated as: zM_W_-BS_x_CL_y_. The molar ratio of each component, determined by ^1^H-NMR, is subscripted (x and y) and z indicates the M_W_ range of each series (low, medium and high), which was determined by gel permeation chromatography (GPC).

### 2.3. Nuclear Magnetic Resonance (NMR)

Both ^1^H NMR and ^13^C NMR spectra were recorded on a Bruker AMX-300 NMR instrument at 300.1 and 75.5 MHz, respectively. TMS was used as an internal reference and the copolyesters were dissolved in deuterated-chloroform (CDCl_3_). The composition of the ε-caprolactone repeating unit in BS_x_CL_y_ was estimated by ^1^H-NMR spectra from proton resonance integrals of methylenes from succinate and ε-caprolactone units. Additionally, the CL-units and BS-units arrangement distributions of were calculated based on ^13^C NMR signals of different methylene groups.

### 2.4. Gel Permeation Chromatography (GPC)

Molecular weight were determined by gel permeation chromatography (GPC) at 35 °C in a Waters equipment, which was calibrated with monodisperse poly(methyl methacrylate) (PMMA) standards. Chromatography was performed with sodium trifluoroacetate-hexafluoroisopropanol (0.05 M) using a poly (styrene-co-divinylbenzene) column at a constant flow rate (0.5 mL/min).

### 2.5. Differential Scanning Calorimetry (DSC)

A Perkin Elmer 8500 calorimeter was used to perform non-isothermal differential scanning calorimetry experiments. The calorimeter was connected to a refrigerated cooling system (Intracooler-2P) with a constant nitrogen flow rate (20 mL/min) and calibrated with pure indium and tin standards. Approximately 5 mg of sample were sealed in standard aluminum pans. The experiments were performed by the following protocol: Firstly, polymer samples were heated up from room temperature (around 25 °C) to 30 °C above their melting point (*T_m_* + 30 °C) and held at this temperature for 3 min to erase any thermal history of the polymer. Secondly, samples were cooled down to −60 °C, and finally, they were reheated up to 30 °C above their *T_m_* at 10 °C/min. To define the glass transition temperature (*T_g_*), the thermal history of the samples was erased at 145 °C for BS-rich samples and 90 °C for CL-rich samples, by holding them at this temperature for 3 min. Then, samples were cooled very fast (at about 160 °C/min) to −90 °C, by using the ballistic-cooling option of DSC 8500. Finally, samples were heated at a rate of 20 °C/min. *T_g_* values were calculated from the heating scans at the midpoint of the heat capacity change.

### 2.6. Tensile Test

An Instron 5569 universal testing machine was used to perform the tensile tests. Dumbbell-shaped test specimens with dimensions according to ASTM D-638 (type V, thickness of 200 micrometers) were die-cut from films prepared by hot-pressing using a Collin P200E press. Young’s modulus (*E*), tensile strength (*σ_b_*) and ductility, measured as the elongation at break (*ε_b_*), were calculated from the load-displacement curves obtained at a crosshead speed of 10 mm/min. Five dumbbell-shaped samples were tested for each reported value.

### 2.7. Dynamic Mechanical Thermal Analysis (DMTA)

The phase behavior of copolymers was studied by DMTA (TA Q800 viscoelastometer). Samples were tested in the single cantilever bending mode at a constant heating rate and frequency (4 °C/min, 1 Hz) from −100 °C to 150 °C.

## 3. Results

### 3.1. Synthesis Results

Table S1 shows molar composition, molecular weight and microstructural data for the synthesized copolyesters. NMR results prove/confirm the chemical structure of the random copolyesters. ^1^H and ^13^C NMR spectra of the HM_W_-BS_51_CL_49_ composition are shown in Figure S1 and the spectra of the entire HM_W_-BS_x_CL_y_ series are compared in Figure S2. The content of the ε-caprolactone and butylene succinate repeating units was estimated from methylene proton resonance integrals of CH_2_ (labeled as 1 in Figure S1a) and CH_2_ (labeled as 4 in Figure S1a). In addition, the sequence distributions of the BS and CL repeating units were calculated based on ^13^C NMR signals of the methylene group CH_2_ labeled as b in Figure S1b.

**Figure 2:**
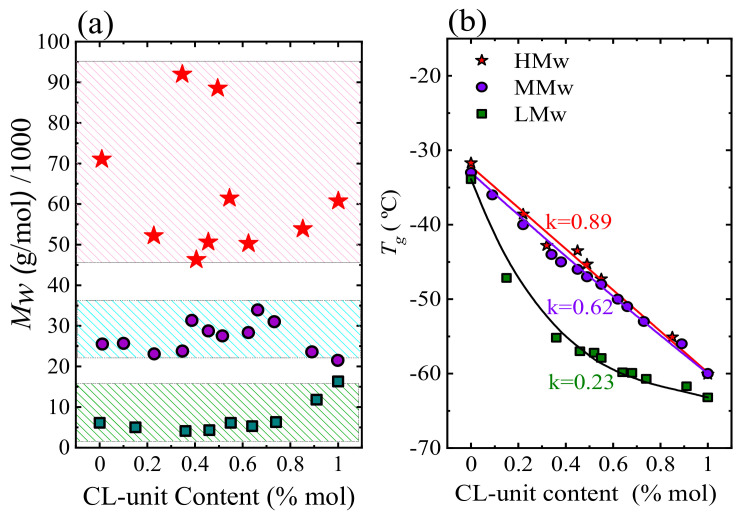
(**a**) M_W_ of samples tested. (**b**) Glass transition temperature values versus composition for three series of copolyesters fitted with the Gordon-Taylor equation. k values are the Gordon-Taylor parameters.

Figure 2a shows the molecular weight versus the CL-unit content of three series of synthesized PBS-*ran*-PCL copolyesters. The *T_g_* values of these 3 series of copolymers are plotted as a function of copolyester composition in Figure 2b. As expected for random copolymers, all copolymers showed a single glass transition temperature, which corresponds to a miscible amorphous phase. The *T_g_* values of the copolyesters are located between the *T_g_* values of the two parental homopolymers and are dependent on composition. Figure 2b shows that the composition dependence follows the Gordon-Taylor fitting equation with the following Gordon-Taylor parameters: *k* = 0.23 for LM_W_, *k* = 0.62 for MM_W_ and *k* = 0.89 for HM_W_-BS_x_CL_y_ copolymers, respectively. These results are consistent with the properties of the random copolymers, as was previously demonstrated by NMR for all series of copolyesters.

### 3.2. Non-Isothermal Melting-Crystallization Behavior

**Figure 3:**
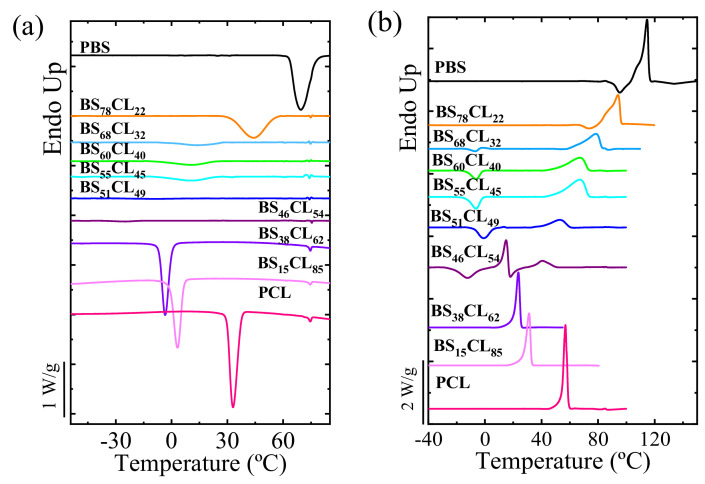
DSC cooling (**a**) and second heating (**b**) runs at a rate of 10 °C/min for the HM_W_-BS_x_CL_y_ copolyesters.

The thermal properties of the synthesized copolyesters of different molecular weights were studied by non-isothermal DSC experiments. The standard non-isothermal behavior of neat HM_W_-PBS and HM_W_-PCL homopolymers, as well as HM_W_-BS_x_CL_y_ copolymers at a cooling and heating rate of 10 °C/min are shown in Figure 3. Table S2 shows the data extracted from DSC traces of HM_W_-copolyesters.

As can be seen in Figure 3a, all copolyester compositions crystallized, and their *T_c_* values were found to be a strong function of their composition. At the scale used in Figure 3, the crystallization peaks for four of the copolyesters were not very sharp. Therefore, a magnified scale of these DSC curves is plotted in Figure S3. When the parental comonomer units have very similar chemical structures, the copolymers can crystallize over the entire composition range.

**Figure 4:**
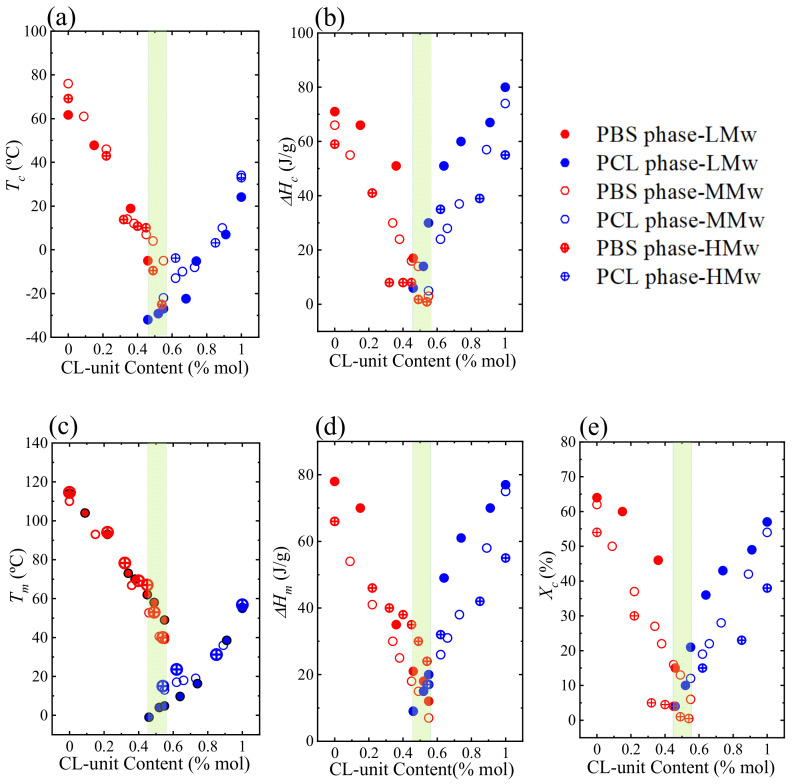
(**a**) Crystallization temperature (*T_c_*); (**b**) crystallization enthalpy (Δ*H_c_*); (**c**) melting temperature (*T_m_*); (**d**) melting enthalpy (Δ*H_m_*); and (**e**) crystallization degree (*X_c_*) values of the three sets of copolyesters. The shaded composition range is the pseudo-eutectic region.

Figure 4a–d show plots of crystallization, melting and the corresponding enthalpy peaks for all 3 sets of copolyesters as a function of their composition. Figure 4a,c show that both crystallization and melting temperatures of the copolymers are controlled by the composition. The effect of the molecular weight on both crystallization and melting temperature is very small (in some cases a small increase with molecular weight is appreciated). Firstly, it should be considered that all materials synthesized possess molecular weights higher than the critical value for the development of entanglements, and are therefore close to molecular weight values where these first order transitions are independent of molecular weights. Secondly, *T_c_* and *T_m_* values in isodimorphic random copolymers are regulated by the randomness of the distribution of the comonomer units and their arrangement through crystallization. In fact, the *T_m_* values are governed by the lamellar thickness achieved. However, both the crystallinity degree and the *T_g_* values of these isodimorphic random copolymers did change significantly with molecular weight, as these parameters depend on chain mobility and amorphous regions chain dynamics, respectively.

Figure 4b,d shows the dependence of crystallization enthalpy (Δ*H_c_*) and enthalpy of melting on the copolymer composition, respectively. The degree of crystallinity (*X_c_*) values of the copolyesters were calculated from the normalized melting enthalpies (normalized by their composition) and were plotted in Figure 4e. Both Figure 4d,e show the enthalpy of melting and the degree of crystallinity displayed a pseudo-eutectic-like behavior. The reduction in crystallinity of the phases as their composition approached the pseudo-eutectic point is caused by the comonomer exclusion during crystallization. As the comonomer content increases for both crystalline phases, comonomer exclusion decreases the length of crystallizable sequences that are included by limiting the number of the second comonomer units. Subsequently, increasing the amount of the second comonomer leads to a decrease in crystallization, because of the higher exclusion of the second comonomer from the crystal lattice.

In addition, Figure 4e shows the dependence of the degree of crystallinity on the molecular weight. The crystallinity degree also shows a pseudo-eutectic-like trend when plotted as a function of composition that arises from the comonomer exclusion during crystallization. The dramatic changes experience by the crystallinity with comonomer composition are ideal to tailor the biodegradation of these copolyesters, as biodegradation rate is inversily proportional to the crystallinity degree.

Furthermore, the lowest molecular weight copolyesters are able to show a higher degree of crystallinity than the other copolymers (Figure 4e), because of the faster diffusion of smaller molecules. Consequently, the higher molecular weight copolyesters, HM_W_-BS_x_CL_y_, showed the lowest crystallinity degree, around 20–30% lower than the MM_W_ copolyesters.

In isodimorphic copolymers, the major comonomer crystallizes within the unit cell of its homopolymer while it incorporates a small amount of the minor comonomer within the crystal lattice. The comonomer exclusion normally dominates when there is a competition between comonomer inclusion and comonomer exclusion. However, comonomer inclusion is essential for the formation of crystals over the whole composition range.

**Figure 5:**
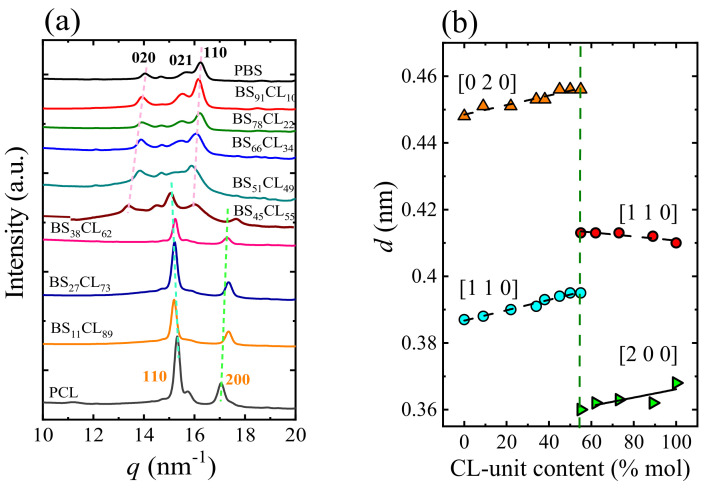
(**a**) WAXS diffraction patterns of MM_W_-BSxCLy recorded at −60 °C after cooling from the melt at a 10 °C/min rate. (**b**) *d*-spacings calculated from the WAXS peaks shown in (**a**) versus composition. Reprinted with permission from ref. [37]. Copyright 2018 American Chemical Society.

Figure 5 shows WAXS diffractogram of MM_W_-copolymers. The characteristic reflections at *q* = 13.9, 15.6 and 16.1 nm^−1^ correspond to the PBS (020), (021) and (110) crystallographic planes and the reflections at *q* = 15.3 and 17.4 nm^−1^ belong to the PCL (110) and (200) planes. Figure 5a shows that on the right side of the pseudo-eutectic point, PCL-like crystals are formed and, on the left side of this point, only PBS-like crystals are detected. For the composition at the eutectic point (MM_W_-BS_45_CL_55_), both crystalline phases were observed. As can be seen in Figure 5b, the *d*-spacings values of both PBS-rich and PCL-rich crystalline phases display important variations with comonomer content that are related to the changes in unit cell sizes and proves that there is comonomer inclusion.

The pseudo-eutectic sample, MM_W_-BS_45_CL_55_, exhibits a very particular phase behavior, one or both phases can be formed depending on the crystallization conditions (via nonisothermal or isothermal crystallization) [37,38].

**Figure 6:**
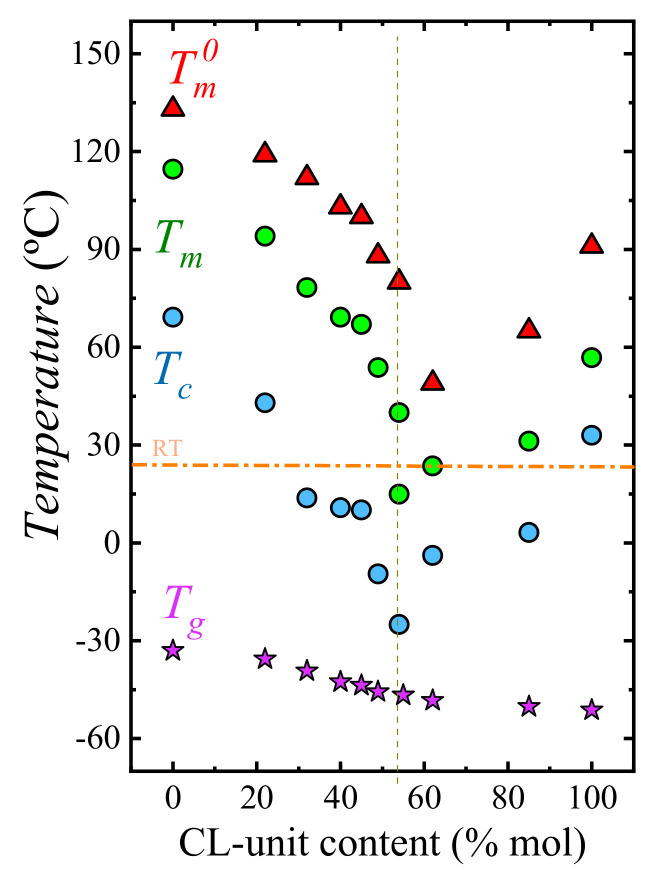
Phase diagram of the HM_W_-BS_x_CL_y_ copolymers based on the non-isothermal crystallization, also showing equilibrium-melting temperatures (*T_m_*^0^) obtained by the Hoffman-Weeks analysis of the isothermal crystallization of samples. The short-dashed upright line specifies the pseudo-eutectic point. The dash-dotted horizontal line represents the room temperature.

Figure 6 shows the phase diagram of HM_W_-BS_x_CL_y_ copolyesters. This series of random high-molecular-weight copolymers display similar calorimetric properties to medium-molecular-weight BS_x_CL_y_ [37]. They show a single-phase melt and a single glass transition temperature and, upon cooling from the melt, the materials crystallize over the entire composition range as confirmed by *X*-ray diffraction studies. The copolymers display a pseudo-eutectic point, including two *T_m_* values at the HM_W_-BS_46_CL_54_ composition, where two crystalline phases can form upon cooling from the melt, as proved earlier by WAXS and DSC. To each side of the pseudo-eutectic point, a single crystalline phase is formed; the left-hand side of the eutectic point is dominated by the PBS-rich phase and the right side, by the PCL-rich phase. It is interesting to note how one can obtain a separate control over *T_g_* and *T_m_* by regulating the composition of the copolymers. This affords unprecedented control to tailor the properties of these fascinating materials.

### 3.3. Controlling Single or Double Crystalline Phases at the Pseudo-Eutectic Point by Non-Isothermal and Isothermal Crystallization

**Figure 7:**
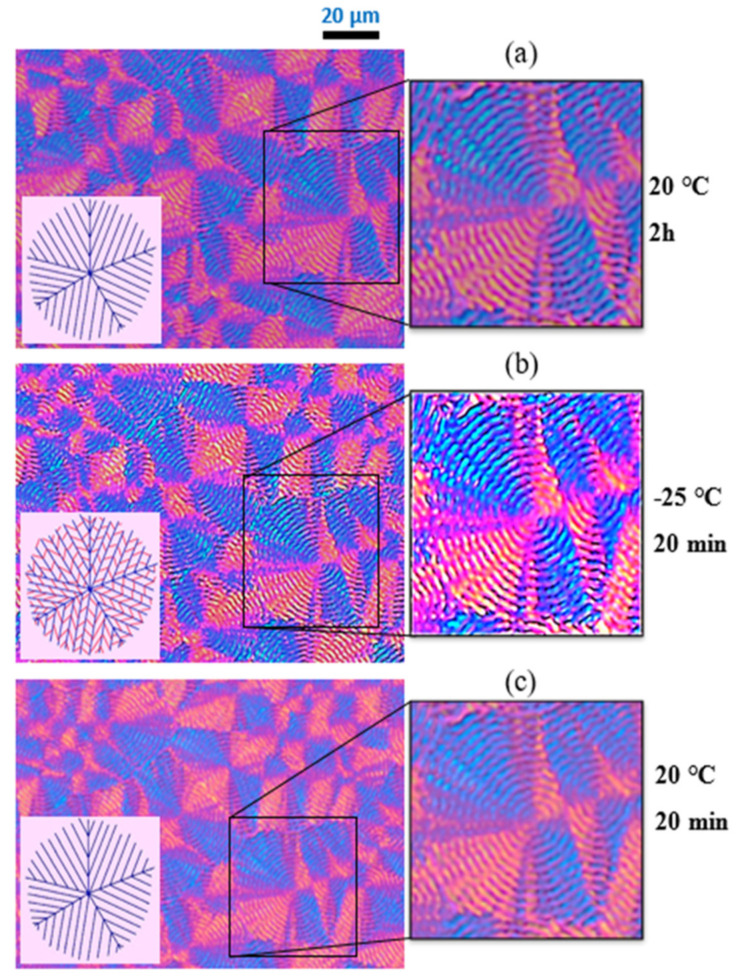
PLOM micrographs for the sample at the pseudo-eutectic point (MM_W_-BS_45_CL_55_). The schematic models are inserted at the bottom left of the PLOM images: (**a**) Crystallization of the sample by fast cooling from the melt to 20 °C (holding for 2 h). (**b**) Second step of crystallization by fast cooling from 20 °C to −25 °C (holding for 20 min). (**c**) Heating the sample to 20 °C at a 10 °C/min rate. The blue lines in the schematic models display BS-rich lamellae, while the red lines represent CL-rich lamellae and the amorphous phase is represented by the pink background. Reprinted with permission from ref. [37]. Copyright 2018 American Chemical Society.

Of the MM_w_ random copolyesters, only the MM_W_-BS_45_CL_55_ composition shows a double crystalline morphology. This copolyester was isothermally crystallized from the melt at 20 °C for 2 h (see Figure 7a). At this temperature, when cooling from a single molten phase, firstly the PBS-rich phase (blue lamellae in schematic diagrams) forms the spherulitic templates and the PCL chains are in the amorphous interlamellar regions.

Quenching the sample from 20 °C to −25 °C results in the crystallization of the PCL-rich phase (red inner lamellae in the sketch) within the intraspherulitic region of the PBS-rich phase (see Figure 7b). Therefore, in Figure 7b, spherulite birefringence increases and the spherulitic structures look brighter, due to the double crystalline structure. Reheating the sample to 20 °C causes the PCL-rich phase to melt from the intraspherulitic regions of the PBS phase while the PBS-rich phase spherulites remain in their crystalline form (Figure 7c).

### 3.4. Controlling the Crystalline Phase over Non-Isothermal and Isothermal Crystallization for Compositions at the Pseudo-Eutectic Point

**Figure 8:**
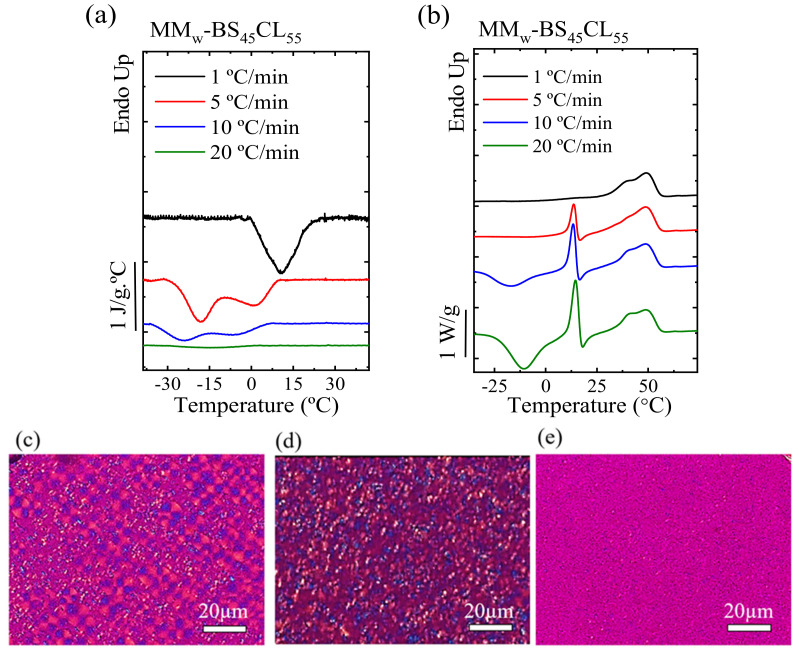

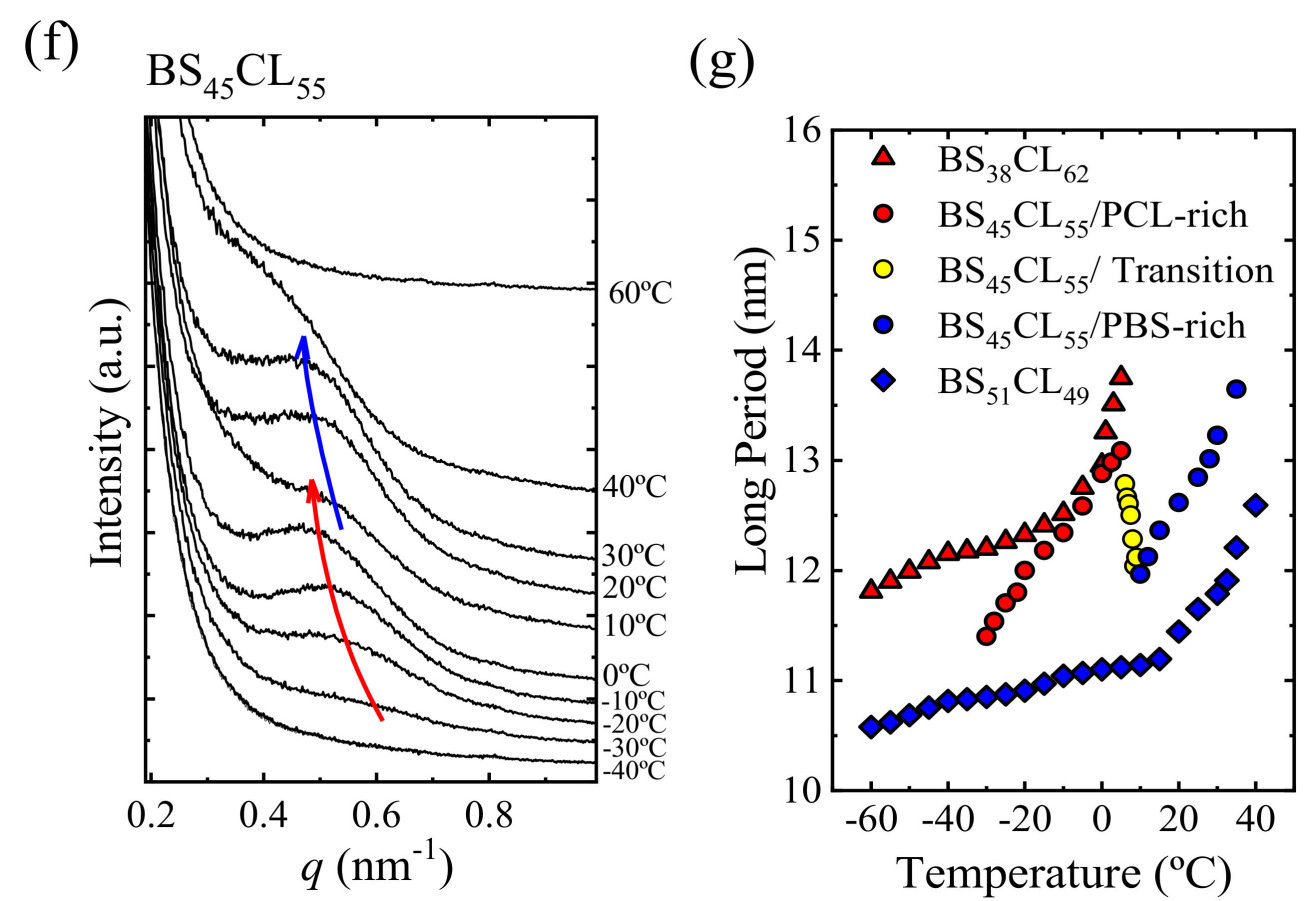
For MM_W_-BS_45_CL_55_ sample (the sample at the pseudo-eutectic point): (**a**) cooling runs from the melt to −60 °C at different cooling rates, (**b**) subsequent heating scans at 20 °C/min. (**c**) PLOM micrographs taken after non-isothermal crystallization at different cooling rates: 5 °C/min, (**d**) 10 °C/min and (**e**) 20 °C/min. (**f**) SAXS patterns registered during heating at 10 °C/min. (**g**) Long period values extracted from the SAXS maxima during cooling for compositions close to and at the pseudo-eutectic point. Reprinted with permission from ref. [37]. Copyright 2018 American Chemical Society.

Figure 8a shows cooling runs of the MM_W_-BS_45_CL_55_ composition at the pseudo-eutectic point at different cooling rates and Figure 8b shows subsequent heating scans at 20 °C/min. The cooling rate has a clear effect on the PBS-rich and PCL-rich crystal phases at the pseudo-eutectic point. At a very low cooling rate (1 °C/min), the PBS-rich phase has enough time to fully grow well-developed spherulites of the PBS-rich phase that hinder the crystallization of the PCL-rich phase due to confinement effects, as can be seen in PLOM observations in Figure 8c, where only PBS-rich crystals can be seen.

When increasing the cooling rate to 5 and 10 °C/min, the DSC cooling scans show a bimodal crystallization exotherm. The DSC evidence clearly indicates that at these cooling rates both PBS and PCL-rich phases are able to crystallize and the PLOM micrograph in Figure 8d shows crystals of both phases.

At higher cooling rates (20 °C/min and faster), the PBS-rich phase cannot crystallize during the cooling process, allowing the PCL-rich phase to develop crystallinity as the PLOM micrograph in Figure 8e shows only very small spherulites of PCL-phase. Upon subsequent heating at 20 °C/min after non-isothermal crystallization, the PCL-rich phase undergoes cold crystallization for samples crystallized at rates above 5 °C /min and its crystallinity content increases.

Figure 8f shows the SAXS patterns that were registered during the heating run for the sample at the pseudo-eutectic point (BS_45_CL_55_ composition). The position of the single peak and the sharpness of the curves change with temperature. There is a remarkable change in the trend of the peak position above 0 °C, as soon as the PCL-rich crystals start to melt and the PBS-rich phase undergoes cold-crystallization.

In Figure 8g, the long period values (obtained from the SAXS maxima) are plotted as a function of temperature for the samples discussed above in Figure 8f. The composition at the pseudo-eutectic point shows a clear transition in the temperature region where the PCL-rich crystals melt and the PBS-rich phase undergoes cold crystallization.

**Figure 9:**
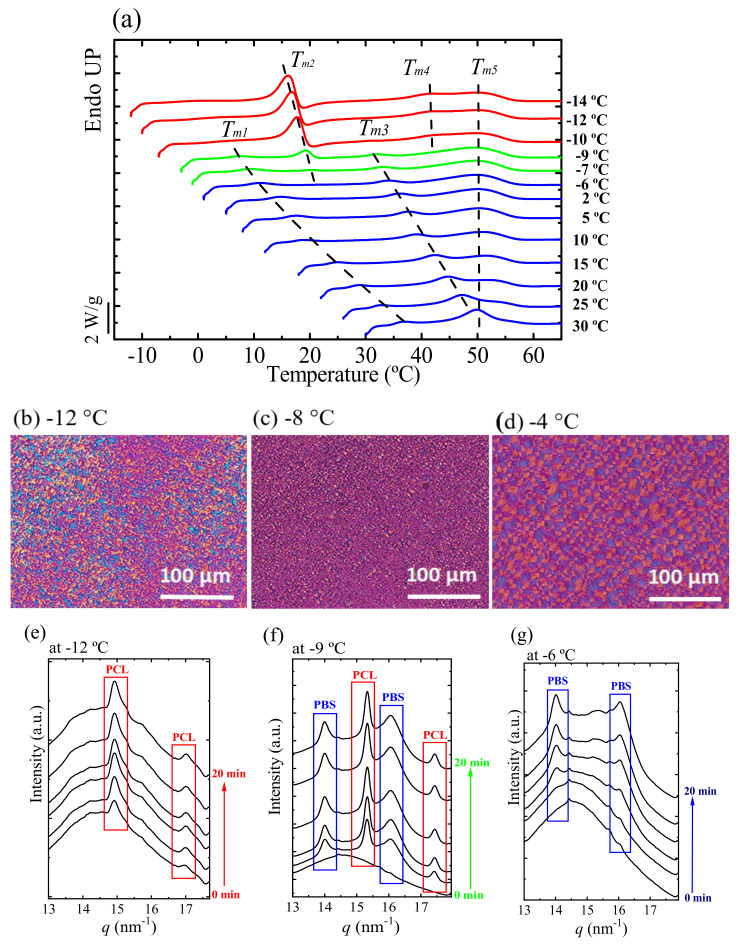
(**a**) DSC heating scans (10 °C/min) after isothermal crystallization at indicated different temperatures for MM_W_-BS_45_CL_55_-copolyester. PLOM micrographs taken after isothermal crystallization at −12 °C (**b**), −9 °C (**c**) and −4 °C (**d**). WAXS patterns of the sample at the pseudo-eutectic point recorded during isothermal crystallization at −12 °C (**e**), −9 °C (**f**) and −6 °C (**g**). See ref. [38] for more details.

In Figure 9a, DSC heating scans are recorded at 10 °C/min for a sample at the pseudo-eutectic point (MM_W_-BS_45_CL_55_) after isothermal crystallization at the indicated *T_c,iso_* values from −14 to 30 °C. The *T_m_*_2_ corresponds to the melting of PCL-rich crystals and it appeared at *T_c,iso_* values lower than −6 °C. The *T_m_*_3_ peak depends on the *T_c,iso_* and relates to the melting temperature of the PBS-rich crystals and it disappeared in the DSC heating curves where the *T_c,iso_* is less than −9 °C.

Figure 9b shows that only small spherulites of PCL crystals were formed at *T_c,iso_* = −12 °C. At *T_c,iso_* = −8 °C, where both PCL and PBS crystals can form, there are crystals of two sizes: PBS crystals with larger spherulites and PCL crystals with smaller spherulites (see Figure 9c). Figure 9d shows only PBS spherulites that were formed at *T_c,iso_* = −4 °C.

Figure 9e–g shows selected WAXS patterns for MM_W_-BS_45_CL_55_ samples that were isothermally crystallized at −12, −9 and −6 °C. Figure 9e shows that at −12 °C only the PCL-rich phase is able to crystalize, although at a higher temperature, −6 °C, only the PBS-rich phase crystallizes (see Figure 9g). On the other side, Figure 9f shows that at the intermediate crystallization temperature, −9 °C, both PBS-rich and PCL-rich phases are able to crystallize.

Bearing in mind the WAXS and DSC results shown in Figure 9, the DSC curves were plotted in color to indicate which phases can crystallize depending on the isothermal crystallization temperature. For the *T_c,iso_* values at −10 °C or lower, only the PCL-rich phase can form (see red curves in Figure 9a). When the *T_c,iso_* values are between −9 °C and −7 °C, both the PCL-rich and the PBS-rich phases are able to crystallize (see green curves in Figure 9a). Finally, if the *T_c_* temperatures are −6 °C and above, only the PBS-rich phase can form (see blue curves).

According to the isothermal and nonisothermal studies, the properties of this isodimorphic copolyester at the pseudo-eutectic point can be tailored by controlling the crystallization conditions.

### 3.5. Isothermal Crystallization

#### 3.5.1. Nucleation Kinetics Studied by PLOM

**Figure 10:**
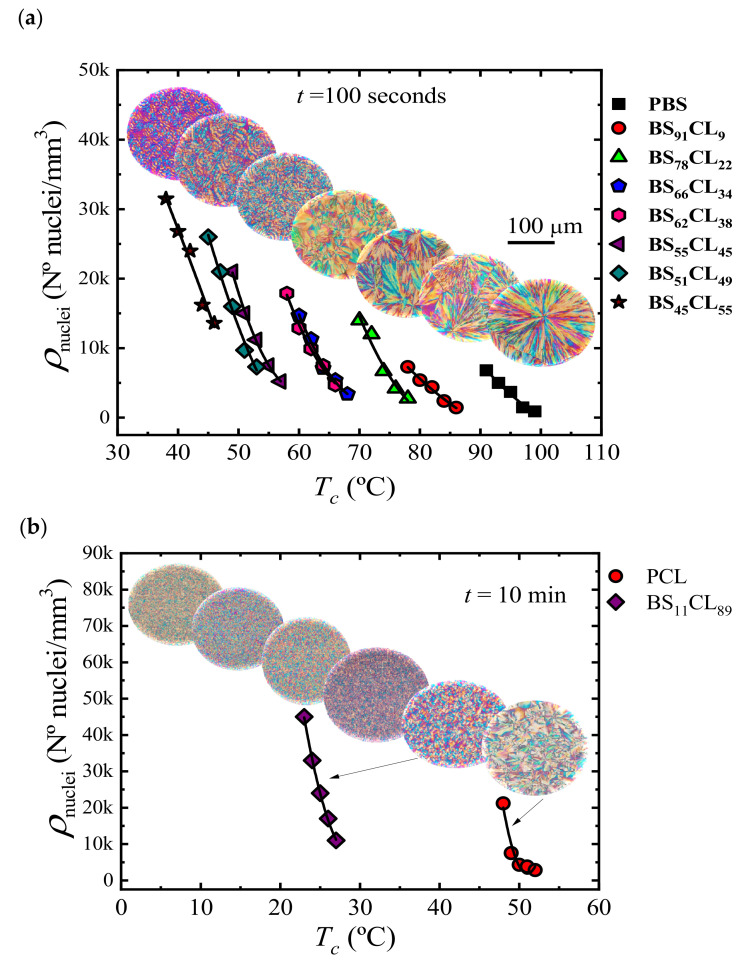
Nuclei density (ρ nuclei) as a function of crystallization temperature (*T_c_*) at 100 s for MM_W_-PBS-rich (**a**) and at 10 min of isothermal crystallization for MM_W_-PCL-rich (**b**) compositions. The inserted images are PLOM micrographs of each composition that crystallized isothermally at Δ*T* = 40 °C. Data reported in ref. [38].

Nucleation kinetics for MM_W_-BS_x_CL_y_ copolyesters were studied by determining the nucleation density using PLOM. Figure 10 shows plots of nucleation density versus temperature taken at a constant nucleation time, *t* = 100 s, for neat MMw-PBS and MM_W_-PBS-rich copolymers and *t* = 10 min for neat MM_W_-PCL and MM_W_-PCL-rich copolymers. PLOM micrographs inserted in Figure 10 were taken after isothermal crystallization at a constant supercooling, Δ*T* = 40 °C, for all samples.

PBS exhibits the lowest nucleation density of all samples and, hence, the largest spherulites, as can be seen in the inserted PLOM micrographs in Figure 10a. As the amount of CL comonomer increases in the PBS-rich copolymers, the nucleation density increases, as well as the supercooling needed for nucleation. PLOM micrographs inserted in Figure 10a shows that both spherulitic size and nucleation density are affected by copolymerization; increasing the amount of CL-unit content in the BS-rich copolymers leads to an increase in nucleation density and a reduction in spherulitic size.

On the other side, at an equal supercooling value, PCL shows a higher nucleation density than PBS (see Figure 10a,b). When a small amount of the BS comonomer is randomly incorporated in BS_11_CL_89_ samples, the nucleation density increases considerably. In obvious similarity (see micrographs inserted in Figure 10b), increasing the PBS content in the copolymer causes a rise in the nucleation density and, hence, a reduction in spherulitic size.

**Figure 11:**
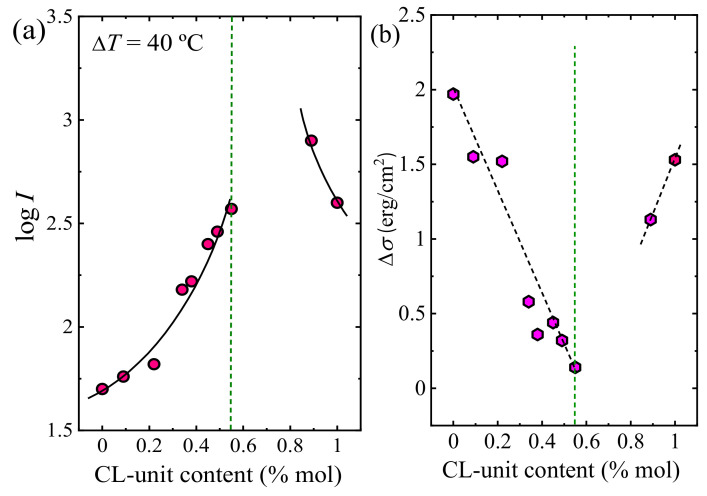
(**a**) Log *I* (nucleation rate) as a function of copolymer composition taken at a constant supercooling of Δ*T* = 40 °C for MMw copolymers. (**b**) Interfacial free energy difference (Δ*σ*) values as a function of CL-unit composition. The dashed vertical lines indicate the pseudo-eutectic composition. Data reported in ref. [38].

The nucleation rate (*I*) values were obtained from the initial slope of the nuclei number versus time plots. As can be seen in Figure 11a, the nucleation rate is strongly influenced by the copolymer composition. The nucleation rate largely increases by adding a comonomer randomly along the chain to either PBS or PCL. As can be seen at the right of the pseudo-eutectic point in Figure 11a, the PCL homopolymer shows faster nucleation than the PBS homopolymer, and the nucleation rate rises by adding PBS copolymer.

Small values of the interfacial free energy difference (Δ*σ*) that were calculated from the Turnbull-Fisher plots, suggested a good nucleation efficiency as lower interfacial energy is necessary to form the crystal–substrate interface. As seen in Figure 11b, copolymerization of PBS with PCL facilitated the primary nucleation process, since the interfacial free energy difference decreases as the amount of CL in the copolymers increases.

At equivalent supercooling, Δ*T* = 40 °C, PCL shows a larger nucleation density than PBS, since PCL has a smaller Δ*σ* value than PBS (see Figure 11b). On the right-hand side of the pseudo-eutectic point in Figure 11b, the Δ*σ* value decreases by the incorporation of 11% of PBS to the copolymer (BS_11_CL_89_ copolymer) as a result of the increase in the nucleation capacity.

**Figure 12:**
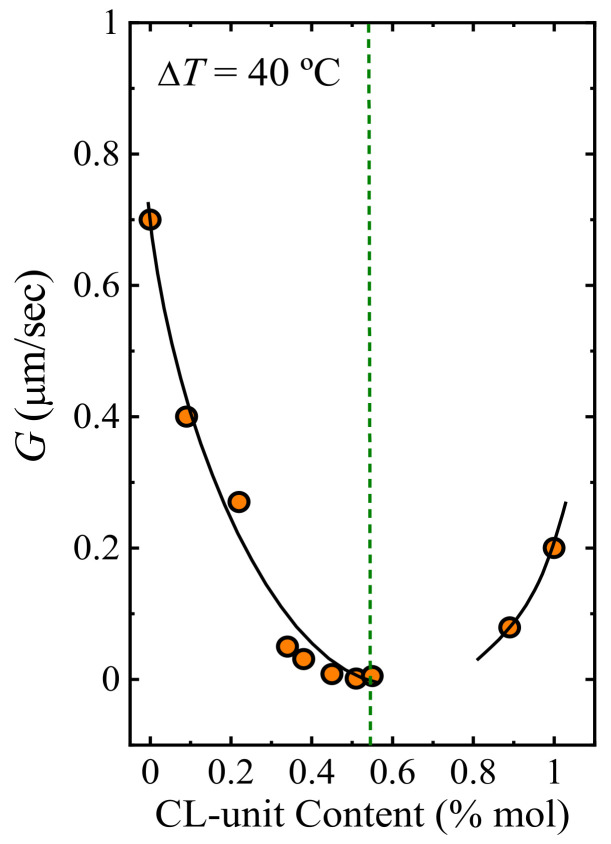
*G* values versus CL-unit content at a constant super cooling of 40 °C. The black lines are polynomial fits. Data reported in ref. [38].

At a constant supercooling of 40 °C, the experimental growth rate values (*G*) decrease with comonomer incorporation for both BS-rich and CL-rich compositions. The reason could be the competition between inclusion and exclusion of repeating units within the crystal lattice, where the exclusion of the second comonomer dominates. Primary nucleation (Figure 11a) and secondary nucleation (Figure 12) show opposite trends when samples are crystallized at the same supercooling.

#### 3.5.2. Studying Overall Crystallization Kinetics by DSC

Isothermal crystallization was used to study overall crystallization kinetics (a kinetic process that encompasses nucleation and growth).

**Figure 13:**
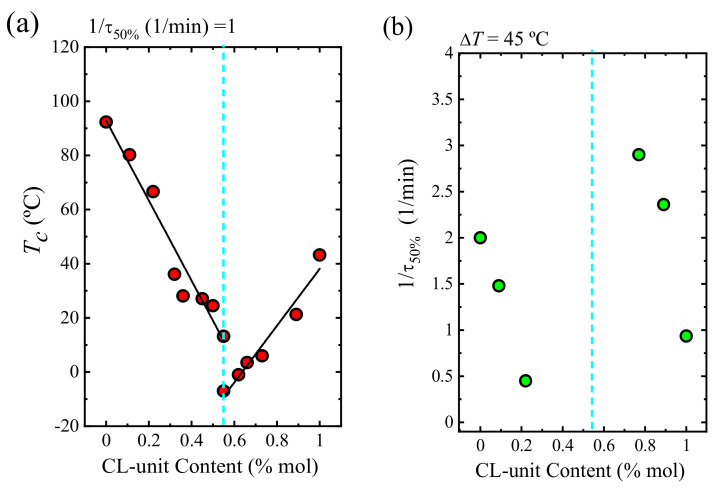
(**a**) *T_c_* values as a function of CL-unit content at a constant rate (1/*τ*_50%_ = 1 1/min). (**b**) Inverse of half-crystallization time (1/*τ*_50%_) as a function of the CL-unit content at a constant degree of supercooling (Δ*T* = 45 °C). Data reported in ref. [38].

As can be seen in Figure 13a, the crystallization temperature tends to decrease as the comonomer content in the copolymer increases until the pseudo-eutectic point is reached at a constant crystallization rate of 1 min^−1^. This result is surely related to the plasticization effect caused by the molten PCL component. Moreover, the PCL exclusion from the PBS-rich crystal lattice may cause a greater reduction of the crystallization rate.

Figure 13b shows the overall crystallization rate versus composition at constant supercooling (45 °C). For PBS-rich compositions, the overall crystallization rate decreases with the addition of CL units. A comparison with Figure 11a and Figure 12 shows that the growth rate controls the overall crystallization of the PBS-rich compositions. Figure 13b shows that at the right-hand side of the pseudo-eutectic point, the acceleration in the overall crystallization rate by the addition of BS comonomer can be explained by the increase in both primary and secondary nucleation rates.

**Figure 14:**
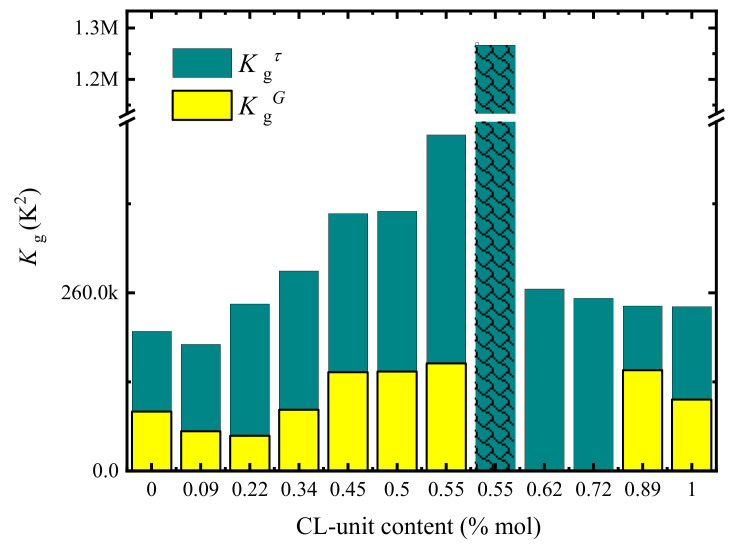
*K_g_* values versus copolymer composition calculated from PLOM experiments (*K_g_^G^*) and DSC experiments (*K_g_^τ^*) for MM_W_-BS_x_CL_y_ samples. This plot was reported in ref. [38].

The *K_g_^G^* values were obtained by applying the Lauritzen-Hoffman theory, and are proportional to the energy barrier for crystallization. Figure 14 plots both *K_g_* values as a function of the CL-unit molar content. The *K_g_^G^* values are obtained from PLOM studies and are related to the energy barrier for spherulitic growth, and *K_g_^τ^* values are obtained from DSC studies and are related to the energy barrier for both primary nucleation and spherulitic growth. As expected *K_g_^τ^* exhibit larger values (as they include both nucleation and growth) than *K_g_^G^* values which depend on growth only.

On the PBS-rich side, *K_g_^G^* and *K_g_^τ^* values increased with the CL-unit molar content, since the growth rate decreased and the nucleation rate increased with comonomer incorporation. On the PCL-rich side, where nucleation and growth rates show opposite trends, there is a counterbalance between nucleation and growth, which causes the *K_g_^τ^* values to remain almost constant with composition. These results prove that there is a clear asymmetry depending on which side of the pseudo-eutectic region the composition is.

### 3.6. Mechanical Properties

#### 3.6.1. Tensile Tests

**Figure 15:**
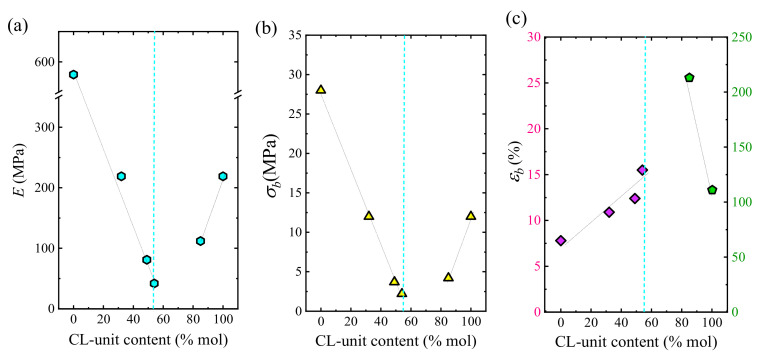
Mechanical properties of HM_W_-BS_x_CL_y_ random copolyesters obtained from stress-strain curves. The elastic modulus *E* (**a**), the stress at break *σ_b_* (**b**) and the elongation at break ε*_b_* (**c**) values are shown versus CL-unit content. The dotted blue line is the pseudo-eutectic composition.

Representative stress–strain curves of the described copolyesters and their parental homopolymers are compared in Figure S4a,b for PBS-rich and PCL-rich phase copolyesters, respectively. Figure 15a–c show, respectively, the Young’s modulus, stress at break and ductility values obtained from these curves plotted against the CL monomer content of the copolymers. As can be seen, the PBS homopolymer shows the highest elastic modulus and stress at break values, and the lowest elongation at break. The introduction of the CL comonomer caused a linear decrease in both *E* and *σ* as compared to the PBS homopolymer in the BS-rich composition range, along with an also linear increase of the elongation at break, with the BS_46_CL_54_ copolymer showing a two-fold increase with respect to PBS homopolymer. This is an expected result given the soft and flexible nature of PCL with respect to the PBS. In fact, a PBS homopolymer with a uniform sequence can exhibit a stronger inter-chain attraction due to its regular structure and the incorporation of flexible CL-units decreases the molecular chain regularity and improves the chain flexibility.

However, it must be noticed that similar behavior can be observed at the other end of the composition range, i.e., in the CL-rich composition range. As can be seen in Figure 15a–c, the incorporation of a small amount of BS comonomer decreased elastic modulus and stress at break and enhanced ductility. The copolymer with 15% PBS showed a close-to-rubbery behavior with a high elongation at break and relatively low elastic modulus. Cao et al. [39] studied the tensile behavior of PBS-*ran*-PCL copolymers with average *M_n_* around 55,000 g/mol and found that the maximum elongation at break occurred for the 18% PBS composition, which is fairly close to the results obtained in this work.

When the behavior of these three mechanical properties is considered as a whole, a clear pseudo-eutectic trend can be observed when they are plotted as a function of the CL-unit content. Jin et al. [40] reported a comparable pseudo-eutectic behavior in those properties for isodimorphic poly (ethylene brassylate-*ran*-δ-valerolactone) copolyesters. This pseudo-eutectic mechanical behavior is similar to that previously described for the degree of crystallinity, *X_c_*, (see Table S2) and points to a non-surprising influence of this parameter on the mechanical properties.

It is worth noting that the degree of crystallinity is not the only factor determining the mechanical properties of the random copolymers. For instance, if we consider BS_68_CL_32_ (*X_c_* = 5%) and BS_15_CL_85_ (*X_c_* = 23%), the copolymer containing BS-rich sequences presented higher *E* and *σ_b_* with a poorer *ε_b_*, despite the lower degree of crystallinity. As previously mentioned, the higher CL comonomer content in the latter with respect to the former must be considered, but it also agrees with a change in the major phase in these random copolyesters, as previously demonstrated by WAXS. Therefore, the mechanical behavior of isodimorphic random copolyesters are determined by the content of both crystalline and amorphous phases of the copolyesters.

#### 3.6.2. DMTA results

**Figure 16:**
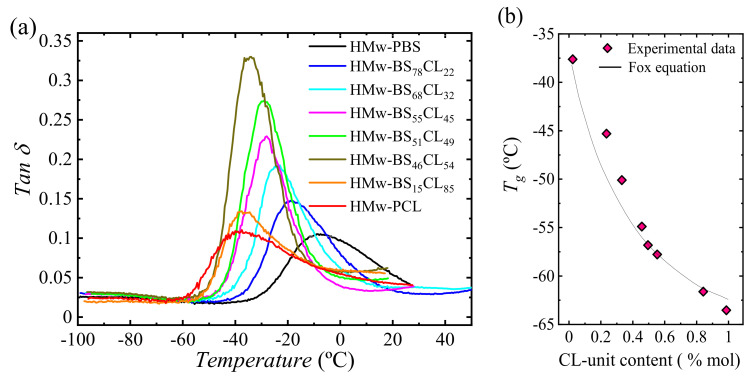
(**a**) Loss tangent (tan *δ*) of the copolyesters as a function of temperature at a constant heating rate (4 °C/min) and frequency (1 Hz). (**b**) *T_g_* values of HM_W_-BS_x_CL_y_ copolyesters (scattered data) as a function of in CL-units content (% molar) and their fitting in the Fox equation (solid black line).

Figure 16a shows the loss factor (tan *δ*) of hot-pressed samples as a function of temperature. The glass transition temperatures (*T_g_*) of copolyesters calculated from the maximum peak in tan *δ* are summarized in Table 1, along with those obtained from the loss modulus (*E*″) peak and DSC, for comparison purposes. As can be observed, the neat PBS sample showed the highest *T_g_* (−10 °C); then, the *T_g_* value shifted to lower temperatures as the amount of CL comonomer was increased. The differences between the *T_g_s* measured by means of the tan *δ* values and the *E*″ values are due to the fact that the tan *δ* peak corresponds to the transition midpoint and the *E*″ peak corresponds to the onset transition region temperature [41].

Typically, the *T_g_* values of the random copolymers are located between the *T_g_* values of two parental homopolymers and strongly rely on their composition [42]. To estimate the *T_g_* dependence on composition for a random copolymer, the Fox Equation (1) is usually applied [43]:

1/*T_g_* = *w*_1_/*T_g_*_1_ + (1 − *w*_1_)/*T_g_*_2_ (1)

where *T_g_*_1_ and *T_g_*_2_ are the glass transition temperatures of corresponding homopolymers 1 and 2 (*T_g_*_1_ < *T_g_*_2_) and *w*_1_ and *w*_2_ are the mass fraction of monomers 1 and 2, respectively. As can be seen in Figure 16b, the composition dependence was found to follow closely the Fox equation (Equation (1)).

As usual, the *T_g_* values obtained from DMTA (Figure 16b and Table 1) were 20 °C higher than those measured by DSC (Figure 2b and Table 1). DMTA applies a heating rate and simultaneously a mechanical deformation at a particular frequency; therefore, this factor increases the rate at which *T_g_* is being measured. The different method and conditions (heating rate) for the measurement in each experimental technique explain these differences.

The maximum intensity of the tan *δ* peak can be applied for the quantitative analysis of the amorphous phase of the polymers [44]. As can be observed in Figure 16a, the intensity of the tan *δ* peak increased at compositions close to the pseudo-eutectic one, pointing to a higher amount of the amorphous phase, in good agreement with previous DSC results. Furthermore, for PBS-*ran*-PCL copolymers, the tan *δ* peak became narrower than in the case of the neat PBS and PCL, which may suggest that chain mobility is higher in the copolymers than in the homopolymers, probably due to the loss in chain regularity.

**Figure 17:**
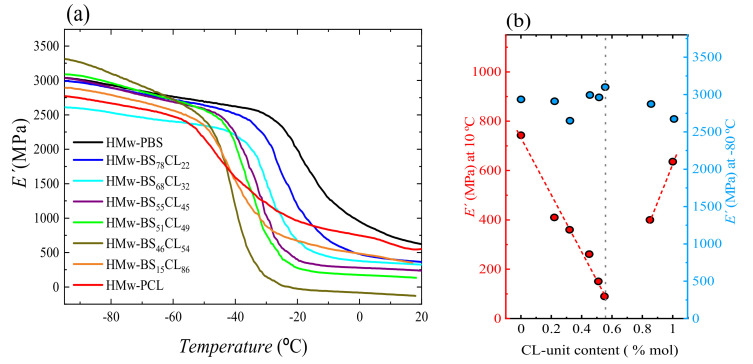
(**a**) Storage modulus of the HM_W_ copolyesters as a function of temperature at 1 Hz. (**b**) Storage modulus values at −80 °C and at 10 °C versus CL-unit content. The dashed vertical line shows the position of the pseudo-eutectic composition.

Figure 17a shows the storage modulus of all the copolymers and their corresponding homopolymers against temperature, while the loss modulus is plotted in Figure S5. As can be seen, *E*′ showed a sharp decrease between −40 to −20 °C, depending on the composition. Figure 17b shows the values of the storage modulus *E*′ in the whole composition range at two specific temperatures: −80 and 10 °C. The storage modulus at the first one, which is below the glass transition temperature of the correspondent material in all cases, did not significantly change with composition, within an error of about ±10%. This is because at this temperature both amorphous and crystalline phases are in a rigid state and, consequently, there is no influence of the degree of crystallinity.

On the contrary, Figure 17b also shows that *E*′ at 10 °C, which is above the *T_g_* of the correspondent material in all cases, showed a pseudo-eutectic behavior, similar to that observed both in *X_c_* and Young’s modulus, pointing to the same relation between degree of crystallinity (isodimorphism) and stiffness of the material measured by the tensile test, which is also performed at temperatures above *T_g_*.

## 4. Conclusions

Biodegradable PBS-*ran*-PCL copolyesters with three different molecular weight ranges (low, medium and high) were successfully synthesized by ring-opening polymerization and polycondensation-copolymerization. These random copolyesters could crystallize over the whole composition range and exhibited a pseudo-eutectic point, as evidenced by calorimetry.

The molecular weight variations did not affect the *T_c_* and *T_m_* values as they are regulated by the randomness of the distribution of the comonomer units. However, both the crystallinity degree and the *T_g_* values of these isodimorphic random copolymers did change significantly with molecular weight, as these parameters depend on chain mobility and long-range order cooperative chain movements.

The addition of comonomers to the major phase at both sides of the eutectic point causes an increase in the nucleation rate and nucleation density, but a decrease in the spherulitic growth rate. For PBS-rich copolymers, the overall crystallization rate was governed by the spherulitic growth rate. In contrast, for PCL-rich copolymers, the nucleation rate was the key main factor in the overall crystallization rate.

Only compositions at the pseudo-eutectic region or point can present double crystallization, and the formation of one or two phases is strongly dependent on the isothermal or non-isothermal crystallization conditions.

Comonomer incorporation at each side of the pseudo-eutectic point caused an increase in the elongation at break, and among the samples, the BS_15_CL_85_ composition displayed the highest elongation at break. In these copolymers, the loss modulus and tan *δ* curves exhibited a single *T_g_* peak that shifted from neat PBS to neat PCL by increasing the CL comonomer content. The composition dependence of the elastic modulus and stress at break values showed a similar pseudo-eutectic behavior at the same eutectic point displayed by crystallinity values, which indicated a significant correlation between the composition in isodimorphic copolymers and their mechanical properties. Storage modulus behaved differently below and above the glass transition temperature. Adding a comonomer randomly along the chain to either PCL or PBS did not affect the storage modulus at temperatures below *T_g_*; however, because the degree of crystallinity is the predominant factor affecting the mechanical properties at temperatures above the glass transition, the storage modulus decreased when becoming closer to the pseudo-eutectic point.

The remarkable change in crystallinity and in first order thermal transitions with composition make these biodegradable random isodimorphic copolyester ideal materials to tailor their physical properties and biodegradation rates depending on the desired specific applications.

## Figures and Tables

**Table 1:** *T_g_* values of HM_W_ PBS-*ran*-PCL copolyesters extracted from the tan *δ* peak and *E*″ of DMTA, and DSC measurements.

Composition	*T_g_* (°C), tan *δ*	*T_g_* (°C), *E*″	*T_g_* (°C), DSC
HM_W_ PBS	−10.0	−16.7	−31.7
HM_W_-BS_78_CL_22_	−18.1	−24.0	−38.6
HM_W_-BS_68_CL_32_	−23.3	−28.6	−42.8
HM_W_-BS_55_CL_45_	−28.5	−33.4	−43.5
HM_W_-BS_51_CL_49_	−30.0	−35.3	−45.3
HM_W_-BS_46_CL_54_	−33.8	−40.9	−47.3
HM_W_-BS_15_CL_85_	−36.8	−41.1	−55.1
HM_W_-PCL	−37.5	−43.3	−60.1

## Data Availability

Data sharing not available.

## Supplementary Materials

The following are available online at https://www.mdpi.com/article/10.3390/polym13142263/s1, Figure S1: ^1^H and ^13^C NMR spectra of HMw-BS_51_CL_49_ as representative of the HM_W_-BS_x_CL_y_ copolyesters series, Figure S2: ^1^H (a) and ^13^C (b) NMR spectra comparison of all HM_W_-BS_x_CL_y_ copolyesters series. Figure S3: Cooling DSC scans from the melt for those compositions that show a broad crystallization peak, Figure S4: Representative stress-strain curves of HM_W_-BS_x_CL_y_ random copolyesters. Figure S5: Loss modulus as a function of temperature at a constant heating rate of 4 °C/min and frequency of 1 Hz for HM_W_-BS_x_CL_y_ copolyesters. Table S1: Synthesis results of the copolymerization of butylene succinate and ε-caprolactone. Table S2: Thermal characterization data of HM_W_-BS_x_CL_y_ copolymer obtained from DSC. Table S3: Nucleation and growth constants obtained from isothermal crystallization kinetics of PBS homopolymer and PBS-rich phase compositions obtained by PLOM measurement. Table S4: Parameters obtained from fitting the DSC data presented in Figure 7a,b to the Lauritzen–Hoffman model.
