# Supplementary material for: Synthesis, Structure, Crystallization and Mechanical Properties of Isodimorphic PBS-ran-PCL Copolyesters
Source: Polymers (Basel). 2021 Jul 9;13(14):2263. doi: 10.3390/polym13142263 (PMC8309441; doi:10.3390/polym13142263)
Supplement: Supplementary file 1 [file polymers-13-02263-s001.zip › polymers-1289291-supplementary.pdf]

Supplementary Materials

Synthesis, Structure, Crystallization and Mechanical Properties of Isodimorphic PBS-*ran*-PCL Copolyesters

Maryam Safari [1], Itziar Otaegi [1], Nora Aramburu [1], Guerrica-echevarria[1], Antxon Martínez de Ilarduya [2], Haritz Sardon [1], Alejandro J. Müller [1,3] *

[1] POLYMAT and Department of Polymers and Advanced Materials: Physics, Chemistry and Technology, Faculty of Chemistry, University of the Basque Country UPV/EHU, Paseo Manuel de Lardizabal, 3, 20018 Donostia-San Sebastián, Spain. maryam.safari@polymat.eu, itziar.otaegi@ehu.eus, nora.aramburu@ehu.eus, gonzalo.gerrika@ehu.eus, haritz.sardon@ehu.eus.

[2] Departament d'Enginyeria Química, Universitat Politècnica de Catalunya, ETSEIB, Diagonal 647, 08028 Barcelona, Spain. antxon.martinez.de.ilarduia@upc.edu.

[3] IKERBASQUE, Basque Foundation for Science, Plaza Euskadi 5, 48009 Bilbao, Spain. alejandrojesus.muller@ehu.es

* Corresponding author: email: alejandrojesus.muller@ehu.es. Tel.: +34 943018191.

Table S1. Synthesis results of the copolymerization of butylene succinate and ε-caprolactone.

Polyester	Composition[a] (BS/CL mol/mol)		Molecular weight[b] (g/mol)			Microstructure[c] (S-centered triads content)			
	Feed	Found	M_n	M_w	*Đ*	BSB	BSCL/CLSB	CLSCL	*R*
HMw-PBS	100/0	100/0	32700	70300	2.2	-	-	-	-
HMw-$BS_{78}CL_{22}$	80/20	78/22	19750	51400	2.6	62	26	12	1.03
HMw-$BS_{68}CL_{34}$	70/30	68/32	34000	91300	2.7	55	28	17	1.00
HMw-$BS_{60}CL_{40}$	60/40	60/40	18400	35500	1.9	44	34	22	1.10
HMw-$BS_{55}CL_{45}$	55/45	55/45	17300	39850	2.3	48	18	34	0.98
HMw-$BS_{51}CL_{49}$	50/50	51/49	23200	87850	3.8	44	14	42	1.00
HMw-$BS_{46}CL_{54}$	45/55	46/54	26000	60700	2.3	40	13	47	1.02
HMw-$BS_{38}CL_{62}$	40/60	38/62	17800	39500	2.2	29	16	55	1.01
HMw-$BS_{15}CL_{85}$	15/90	15/85	26350	53100	2.0	8	14	78	1.00
HMw-PCL	0/100	0/100	31000	60000	1.9	-	-	-	-

[a] Composition of the feed and the resulting polymer as determined by ^{1}H NMR.
[b] Number and weight average molecular weights and dispersities estimated by GPC against PMMA standards.
[c] Copolyester microstructure determined by NMR; R is the degree of randomness which should be 1 for a fully statistical distribution of the comonomeric units.
*Polycondensation were performed under vacuum at 220 °C for around 5h.

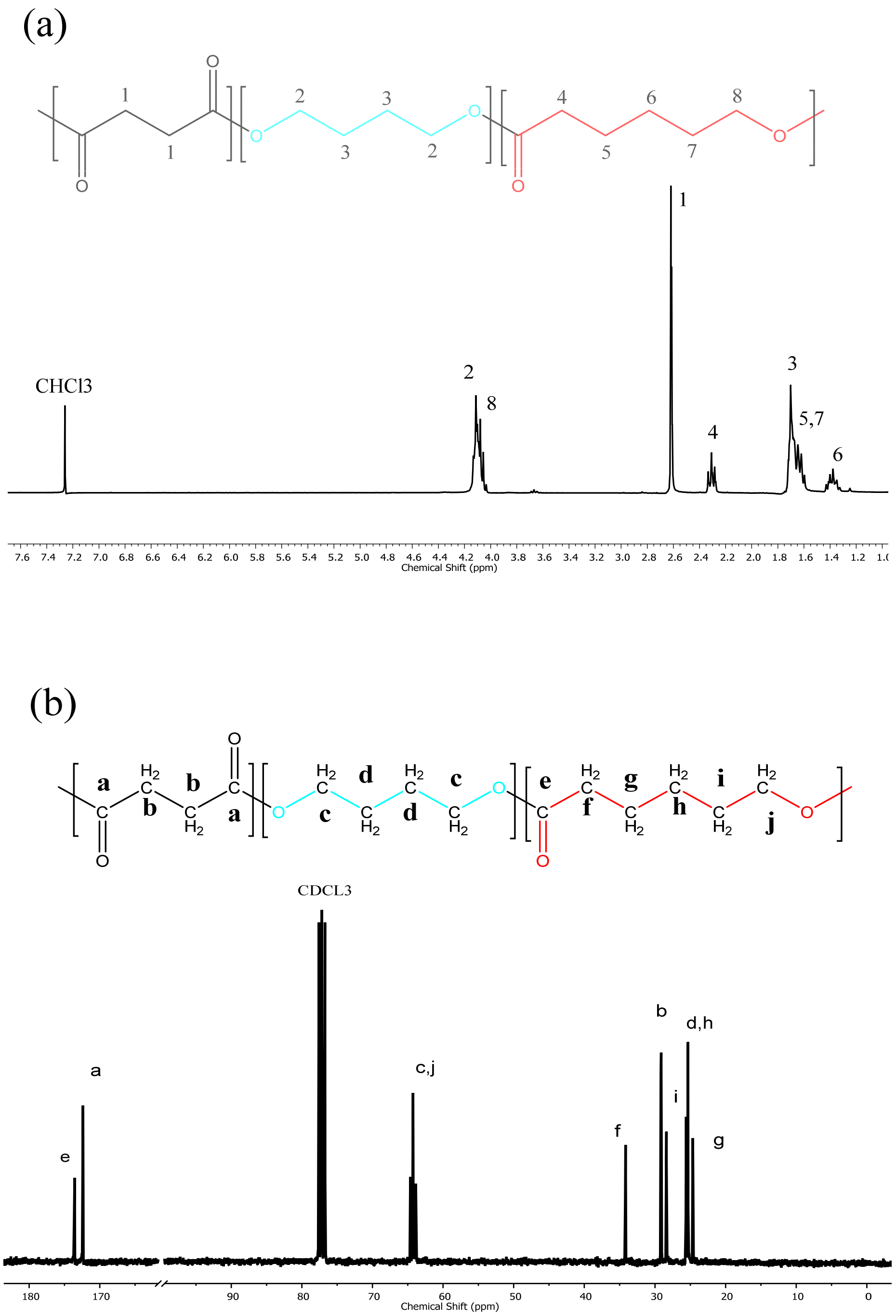

Figure S1. ^{1}H (a) and ^{13}C (b) NMR spectra of HMw-$BS_{51}CL_{49}$ as representative of the HMw-BS_xCL_y copolyesters series recorded in $CDCl_3$. Peaks were assigned according to the expected copolyester constitution depicted in the attached formula.

(a)

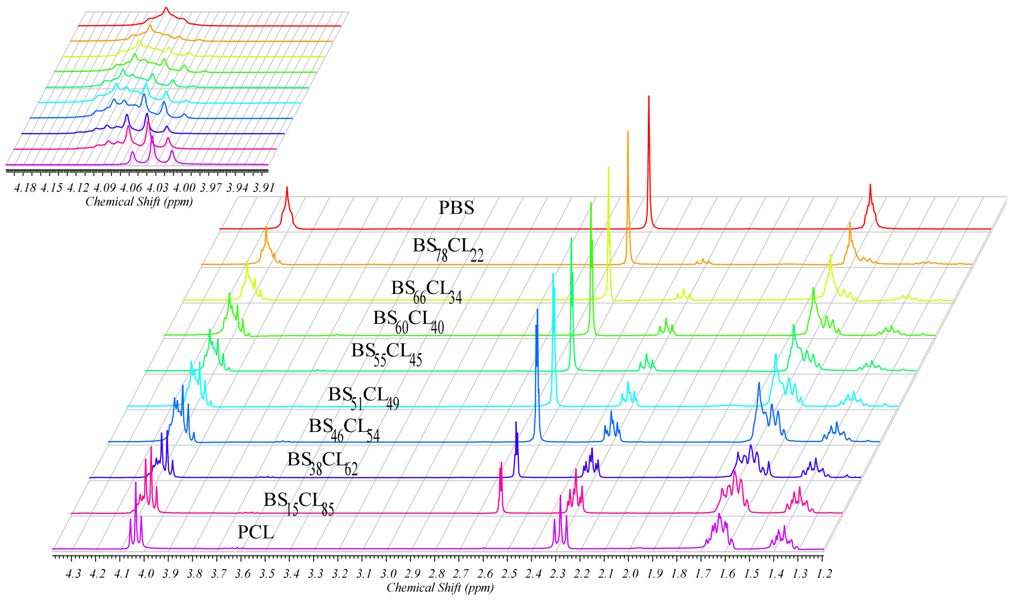

(b)

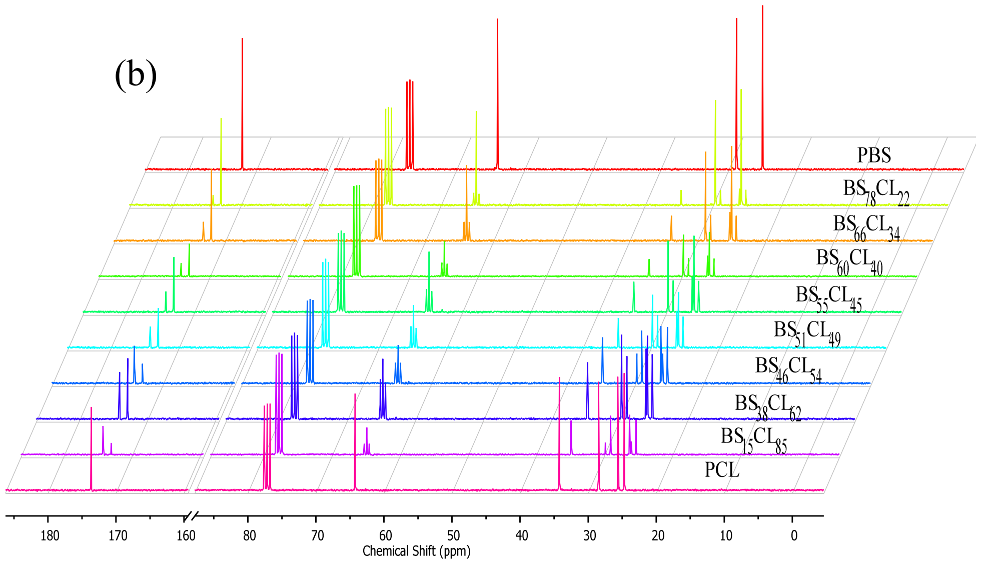

Figure S2. ^{1}H (a) and ^{13}C (b) NMR spectra comparison of all HMw-BS_xCL_y copolyesters series.

Table S2. Thermal characterization data of HMw-BS_xCL_y copolymer obtained from DSC.

Copolymer	T_m (°C)	ΔH_m (J/g)	T_c (°C)	ΔH_c (J/g)	X_c (%)
HMw-PBS	114.6	66	69.2	59	54
HMw-$BS_{78}CL_{22}$	94.1	46	43.0	41	30
HMw-$BS_{68}CL_{32}$	78.3	40	13.8	8	5
HMw-$BS_{60}CL_{40}$	69.2	38	10.8	8	4.5
HMw-$BS_{55}CL_{45}$	67.0	35	10.1	8	4
HMw-$BS_{51}CL_{49}$	53.8	30	-9.5	1.8	1
HMw-$BS_{46}CL_{54}$	40 & 15	17 &24	-25.0	0.9	0.5
HMw-$BS_{38}CL_{62}$	23.6	32	-3.8	35	15
HMw-$BS_{15}CL_{85}$	31.2	42	3.2	39	23
HMw-PCL	56.8	55	33	55	38

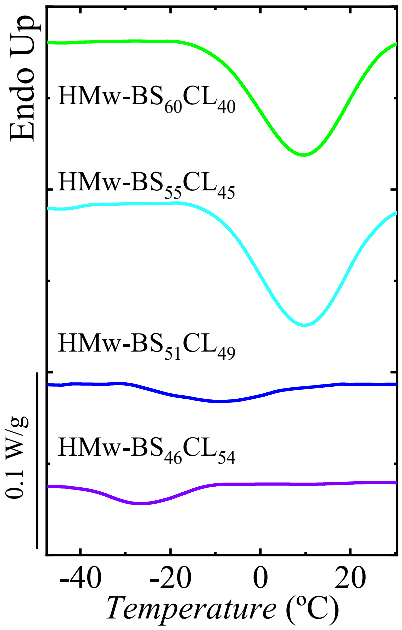

Figure S3. Cooling DSC scans from the melt for those compositions that show a broad crystallization peak.

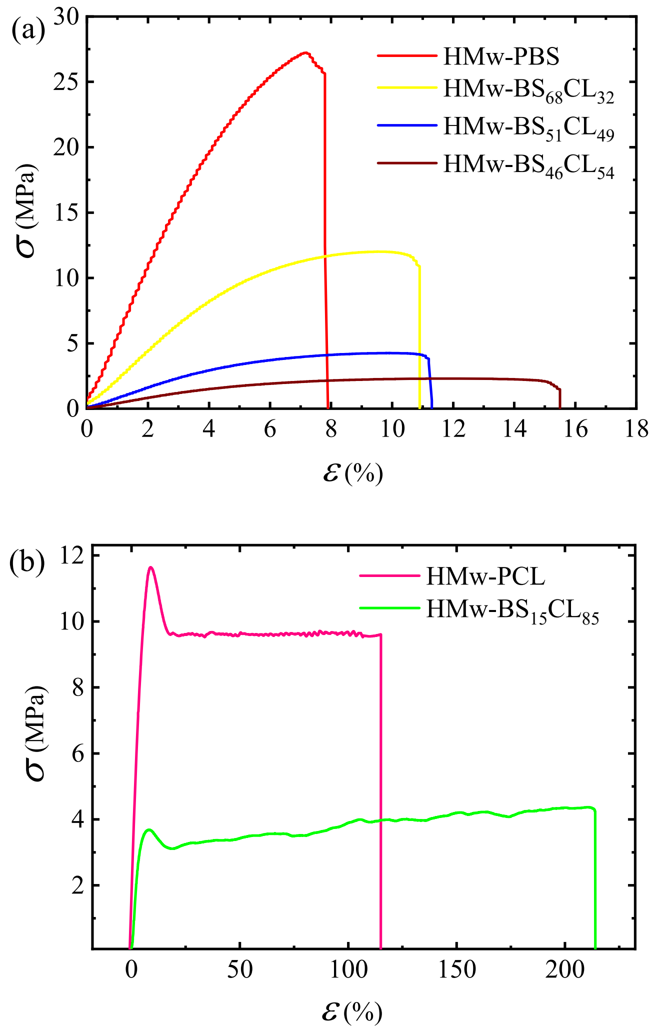

Figure S4. Representative stress-strain curves of (a) BS-rich HMw-BS_xCL_y and (b) CL-rich HMw-BS_xCL_y random copolyesters.

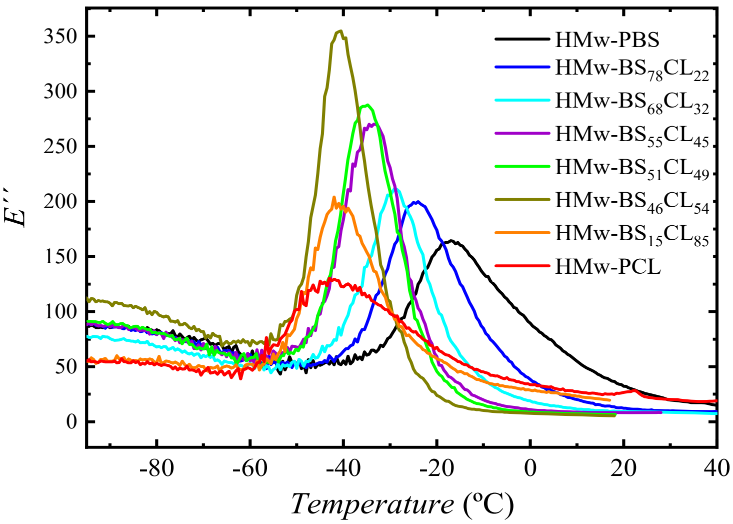

Figure S5. Loss modulus as a function of temperature at a constant heating rate of 4 °C/min and frequency of 1 Hz for HMw-BS_xCL_y copolyesters.

Table S3. Nucleation and growth constants obtained from isothermal crystallization kinetics of PBS homopolymer and PBS-rich phase compositions obtained by PLOM measurement.

Copolyester	K_g^G (K²)	σ (erg/cm²)	σ_e (erg/cm²)	q (erg)	R^2
**PBS**	8.66E+04	12.36	79.52	3.37E-13	0.983
$\mathbf{BS_{91}CL_9}$	5.80E+04	12.36	55.01	2.33E-13	0.994
$\mathbf{BS_{78}CL_{22}}$	5.12E+04	12.36	49.55	2.10E-13	0.973
$\mathbf{BS_{66}CL_{34}}$	8.94E+04	12.36	88.90	3.78E-13	0.982
$\mathbf{BS_{62}CL_{38}}$	8.92E+04	12.36	89.42	3.87E-13	0.982
$\mathbf{BS_{55}CL_{45}}$	14.4E+04	12.36	148.8	6.30E-13	0.999
$\mathbf{BS_{51}CL_{49}}$	14.5E+04	12.36	148.9	6.32E-13	0.999
$\mathbf{BS_{45}CL_{55}}$	15.7E+04	12.36	165.5	7.02E-13	0.974
$\mathbf{BS_{11}CL_{89}}$	14.7E+04	6.83	169.58	6.32E-13	0.999
**PCL**	10.4E+04	6.83	111.97	4.17E-13	0.996

R^2 is the correlation coefficient for the Lauritzen–Hoffman, linear plots, $\ln(1/\tau_{50\%}) + U^*/R(Tc - T\alpha)$ vs. $1/f{\cdot}Tc{\cdot}\Delta T$.

Table S4. Parameters obtained from fitting the DSC data presented in Figure 7 (a-b) to the Lauritzen–Hoffman model.

Copolyester	K_g^t (K^2)	R^2	σ (erg/cm^2)	σ_e (erg/cm^2)	q (erg)
**PBS**	2.04E+05	0.9697	12.36	186.88	7.93E-13
$\mathbf{BS_{91}CL_{9}}$	1.85E+05	0.9938	12.36	173.80	3.37E-13
$\mathbf{BS_{78}CL_{22}}$	2.44E+05	0.9514	12.36	236.34	10.0E-13
$\mathbf{BS_{66}CL_{34}}$	2.92E+05	0.9958	12.36	290.42	12.3E-13
$\mathbf{BS_{55}CL_{45}}$	3.76E+05	0.9775	12.36	387.34	16.4E-13
$\mathbf{BS_{51}CL_{49}}$	3.79E+05	0.9973	12.36	388.50	16.8E-13
$\mathbf{BS_{45}CL_{55}(BS-rich)}$	4.91E+05	0.9502	18.54	518.73	22.0E-13
$\mathbf{BS_{45}CL_{55}(CL-rich)}$	12.5E+05	0.9880	6.83	1376.9	51.3E-13
$\mathbf{BS_{38}CL_{62}}$	2.66E+05	0.9917	6.83	328.01	12.6E-13
$\mathbf{BS_{27}CL_{73}}$	2.52E+05	0.9845	6.83	305.91	11.4E-13
$\mathbf{BS_{11}CL_{89}}$	2.41E+05	0.9945	6.83	278.43	10.4E-13
**PCL**	2.40E+05	0.9082	6.83	260.61	9.71E-13

R^2 is the correlation coefficient for the Lauritzen–Hoffman, linear plots $\ln(1/\tau_{50\%}) + U^*/R(Tc - T\alpha)$ vs. $1/f{\cdot}Tc{\cdot}\Delta T$.
